# RNA‐based medicine: from molecular mechanisms to therapy

**DOI:** 10.15252/embj.2023114760

**Published:** 2023-09-20

**Authors:** Anke Sparmann, Jörg Vogel

**Affiliations:** ^1^ Helmholtz Institute for RNA‐based Infection Research (HIRI), Helmholtz Centre for Infection Research (HZI) Würzburg Germany; ^2^ Institute of Molecular Infection Biology (IMIB) University of Würzburg Würzburg Germany

**Keywords:** antisense oligonucleotide drug, CRISPR‐Cas therapy, mRNA therapeutics, rare genetic disease, RNA interference, Genetics, Gene Therapy & Genetic Disease, Pharmacology & Drug Discovery, RNA Biology

## Abstract

RNA‐based therapeutics have the potential to revolutionize the treatment and prevention of human diseases. While early research faced setbacks, it established the basis for breakthroughs in RNA‐based drug design that culminated in the extraordinarily fast development of mRNA vaccines to combat the COVID‐19 pandemic. We have now reached a pivotal moment where RNA medicines are poised to make a broad impact in the clinic. In this review, we present an overview of different RNA‐based strategies to generate novel therapeutics, including antisense and RNAi‐based mechanisms, mRNA‐based approaches, and CRISPR‐Cas‐mediated genome editing. Using three rare genetic diseases as examples, we highlight the opportunities, but also the challenges to wide‐ranging applications of this class of drugs.

## Graphical abstract

This review discusses the latest research on RNA‐based therapeutics, from design to the clinic.

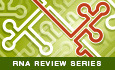

## Introduction

The rapid development of mRNA vaccines in response to the COVID‐19 pandemic has led to a resurgence in interest in RNA as a molecule class in diagnostics, prevention, and treatment of diseases. Whether for infectious diseases, cancer, neurodegeneration, metabolic disorders, or rare genetic diseases, RNA holds great promise for combatting human diseases previously intractable to therapy. Much of this promise builds on the fundamental cellular role of RNA – as a template, catalyst, scaffold, or regulator. The unique structural and biochemical properties of RNA allow it to be targeted or applied in a programmable manner since the rules that govern RNA binding affinity and specificity – complementary base pairing – are well understood. While conventional drug development often involves labor‐ and time‐intensive screening to identify lead compounds, RNA drugs can be rationally designed as long as the target is known. As a result, RNA therapies are emerging as platform technologies that can be applied to a variety of diseases. Moreover, delivery strategies can be developed and optimized independently from the RNA component of the drug, which not only speeds up therapeutic development but also opens the possibility to target ultra‐rare diseases.

Despite its potential, the use of RNA as a therapeutic faces obstacles, including its poor pharmacological properties, difficulty with intracellular delivery, and immune‐related toxicity. Technological advances in medicinal chemistry and a better understanding of natural antisense RNA phenomena were necessary to make RNA drugs a reality. The scientific breakthroughs that revolutionized basic biological research such as the discovery of microRNAs, RNA interference, and CRISPR‐Cas systems (Box 1) are now finding their applications in the clinic.

Box 1 **Relevant discoveries in antisense RNA biology**

The notion of RNA as a regulatory molecule in eukaryotic cells took hold in 1993, when Victor Ambros, Gary Ruvkun and colleagues identified *lin‐4* as a regulator of *Caenorhabditis elegans* development (Lee *et al*, 1993; Wightman *et al*, 1993). Curiously, *lin‐4* did not encode a protein, but expressed a precursor RNA that is processed into a short, double‐stranded RNA. Lin‐4 post‐transcriptionally suppresses Lin‐14, a protein crucial for *C. elegans* larval progression, by recognizing a partially complementary sequence in the 3′ UTR of *lin‐14* mRNA. *lin‐4* is now recognized as the founding member of a family of small regulatory RNAs, the miRNAs. Although it was not until the early 2000s that miRNA‐mediated gene regulation was shown to be evolutionarily conserved and widespread throughout metazoans (Pasquinelli *et al*, 2000), the landmark discovery of *lin‐4* can be seen as a first hint that the cellular functions of RNA are much more complex than simply coding proteins.

Meanwhile, scientists had been observing unexpected RNA‐mediated gene‐silencing phenomena in plants and other experimental systems, including *C. elegans*. Initially, the molecular basis was thought to be an antisense mechanism that depends on hybridization between the regulatory RNA and cellular mRNA transcripts. Then, in 1998, Andrew Fire and Craig Mello showed that, in nematodes, the administration of double‐stranded RNA triggers sequence‐specific mRNA silencing, in a process they coined “RNA interference” (RNAi). Their results argued against stochiometric interference with endogenous mRNA and suggested a catalytic component that amplified the process (Fire *et al*, 1998). This work, and the demonstration that 21‐ and 22‐nt double‐stranded RNAs induce post‐transcriptional gene silencing in plants (Hamilton & Baulcombe, 1999), set the stage for subsequent studies that characterized the molecular mechanism underlying the RNAi pathway. In 2000, two papers reported that the key to RNAi is the conversion of double‐stranded RNAs into small interfering RNAs (siRNAs), which guide an “RNA‐induced silencing complex” (RISC) to enzymatically cleave complementary mRNAs (Hammond *et al*, 2000; Zamore *et al*, 2000; Fig 4). This observation was pivotal to the development of siRNAs that could silence genes in mammalian cells without eliciting innate immune responses (Caplen *et al*, 2001; Elbashir *et al*, 2001). Soon, siRNAs became ubiquitous tools for the targeted inhibition of any gene of interest, based on sequence alone.

The next game changer in RNA biology came with the discovery of an RNA‐guided immune defense system in bacteria and archaea. CRISPR‐Cas systems are now widely known due to their fame as programmable genome‐editing tools. After the initial experimental demonstration that CRISPR‐Cas systems provide adaptive immunity against foreign mobile genetic elements (Barrangou *et al*, 2007) and the identification of the defense mechanism (Garneau *et al*, 2010; Deltcheva *et al*, 2011; Gasiunas *et al*, 2012; Jinek *et al*, 2012), the potential of RNA‐guided Cas nucleases for genome engineering was soon realized. This concept was established in a set of studies published in early 2013, less than 6 months after the demonstration of programmable DNA cleavage by Cas9 (Gasiunas *et al*, 2012; Jinek *et al*, 2012), when *in vivo* proof that RNA‐guided Cas9 could be used to edit genes in both mouse and human cell lines was provided (Cong *et al*, 2013; Jinek *et al*, 2013; Mali *et al*, 2013). This new, easy‐to‐use genome‐editing tool captured the attention of scientists across a wide range of disciplines and the technology rapidly took a key position in biological and biomedical research.

## RNA‐based therapeutic strategies

**Figure 1:**
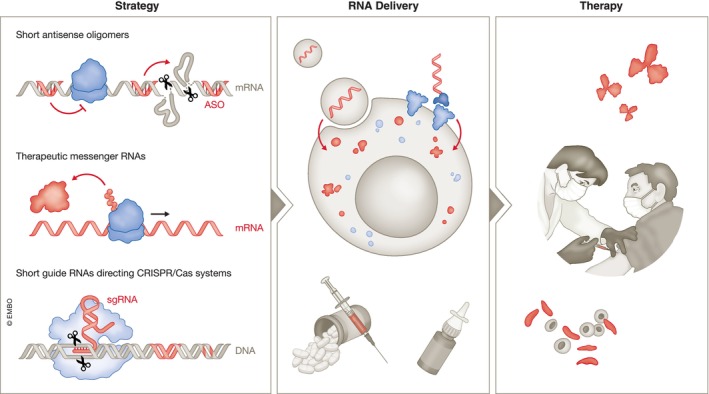
Strategies for RNA‐based medicine Strategies for RNA‐based medicines include antisense oligomers, mRNA‐based approaches and CRISPR‐Cas‐mediated genome editing. Most of these therapeutic modalities require efficient delivery vehicles such as lipid nanoparticles and targeting ligands for therapeutic development.

Conceptually, RNA therapeutics can be divided into three major categories: compounds that target cellular RNA, which are typically heavily modified nucleic acid‐based antisense oligomers; treatments in which RNA itself is the therapeutic agent that is delivered into cells to mediate the expression of a protein; and therapeutic genome editing, in which RNA serves as the guide for an effector protein that modifies the cellular DNA sequence (Fig 1). In this review, we will provide a broad overview of these different strategies with a focus on their molecular modes of action. For more details, we refer the interested reader to recent comprehensive reviews that center on each approach in turn, specifically antisense technologies (Crooke *et al*, 2021a, 2021b; Egli & Manoharan, 2023), RNAi‐based therapeutics (Setten *et al*, 2019), mRNA‐based approaches (Chaudhary *et al*, 2021; Rohner *et al*, 2022) and CRISPR‐Cas‐mediated genome editing (Anzalone *et al*, 2020; Doudna, 2020; Raguram *et al*, 2022; Wang & Doudna, 2023).

### Antisense therapeutics

The quest for antisense‐based therapeutics dates back almost half a century to 1978, when Mary Stephenson and Paul Zamecnik designed a synthetic antisense oligonucleotide (ASO) to inhibit Rous sarcoma virus replication in tissue culture (Stephenson & Zamecnik, 1978; Zamecnik & Stephenson, 1978). These studies pioneered the notion of harnessing the unique chemical properties of nucleic acids for drug design. It took another 20 years until the United States Food and Drug Administration (FDA) approved the first ASO drug for clinical use. This drug – fomivirsen – is a synthetic 21 nucleotide (nt) ASO that binds to a complementary sequence of cytomegalovirus (CMV) mRNA and blocks the translation of proteins essential for CMV replication. It was indicated for the treatment of CMV retinitis, a serious infection of the retina that can lead to blindness, in patients with acquired immune deficiency syndrome (AIDS; Vitravene Study Group, 2002a, 2002b). Despite therapeutic benefits, the drug was withdrawn from the market due to the success of anti‐retroviral therapy. Still, fomivirsen provided the first proof‐of‐concept of the clinical value of ASOs.

Principally, ASOs are short synthetic nucleic acids that bind to cellular RNA through complementary base pairing. RNA itself, in its unmodified form, is rapidly degraded by nucleases, making it unsuitable for use as an ASO drug. Chemical modifications of the nucleosides, nucleobases, and the ASO backbone are therefore essential for drug development. Several classes of nucleic acid analogs with improved stability and resistance to nucleases are currently in use and the field is developing rapidly (Box 2).

Box 2 **Selected backbone, sugar or nucleobase modifications of ASO and siRNA therapeutics**

The goals of oligonucleotide medicinal chemistry are to increase resistance to nucleases, to enhance affinity to the target, to improve pharmacokinetics and to reduce pro‐inflammatory responses. An initial focus lay on modifications of the phosphodiester backbone. In unmodified DNA or RNA oligonucleotides, this backbone is highly susceptible to degradation by nucleases. A major breakthrough was the introduction of phosphorothioate (PS) linkages, in which one of the non‐bridging oxygen in the phosphodiester is replaced by a sulfur atom. This modification increases nuclease resistance, decreases hydrophilicity and promotes the binding of serum proteins, which, in turn, improves circulation life‐time. It also increases binding to cell surface proteins, thereby facilitating ASO uptake into cells (Roberts *et al*, 2020; Crooke *et al*, 2021a). Yet, ASOs with a phosphorothioate backbone retain immunostimulatory activity (Kulkarni *et al*, 2021) and show lower target binding affinity than unmodified ASOs (Freier & Altmann, 1997). 5′‐vinylphosphonate, a metabolically stable phosphate mimic, protects siRNA drugs from phosphatases and improves their silencing activity by enhanced binding to human Argonaute 2 (Ago2; Prakash *et al*, 2015; Parmar *et al*, 2016; Elkayam *et al*, 2017). The modification also increases siRNA accumulation and retention in multiple tissues and extends the duration of silencing *in vivo* (Haraszti *et al*, 2017). PMO morpholinos, which have a backbone of methylenemorpholine rings linked through phosphorodiamidate groups, have improved target affinity and stability, but are uncharged, which decreases serum protein binding and circulation lifetime. They are rapidly eliminated from the body following systemic injection and poorly taken up by cells (Crooke *et al*, 2021a). They also do not activate RNase H1, and therefore rely on regulatory mechanism that are based on steric hindrance (Summerton, 1999). Peptide nucleic acids (PNAs) harbor a pseudo‐peptide backbone that links the four natural nucleobases. This chemical structure also confers resistance toward nucleases and proteases. Like PMOs, PNAs are uncharged, leading to strong binding to complementary sequences, but they face a cellular delivery barrier (Pradeep *et al*, 2023). Other chemical modifications that increase the affinity of oligonucleotides for their targets and thereby improve potency and selectivity center on 2′ ribose modifications, such as 2′‐*O*‐methyl (2′‐*O*‐Me), 2′‐*O*‐methoxyethyl (2′ MOE), and 2′‐fluoro (2′‐F; Kulkarni *et al*, 2021). Full modification is often incompatible with specific mechanisms of actions such as RNase H1 recruitment. Nevertheless, partial modification, especially in flanking sequences, is generally well tolerated. Increased affinity can also be gained by using oligonucleotides modified with locked nucleic acids (LNA), which contain a methylene bridge between the 2′ and 4′ position of the ribose. This bridge secures the ribose ring in a conformation that is ideal for binding to complementary sequences (Braasch & Corey, 2001). Nucleobase modifications are less common, although replacing cytosine with 5‐methylcytosine (5mC) reduces the immunostimulatory effects of ASOs (Henry *et al*, 2000). Introducing these modifications into oligonucleotides is compatible with DNA and RNA synthesis. Therefore, ASOs can be designed to incorporate multiple modifications to combine their advantageous properties.

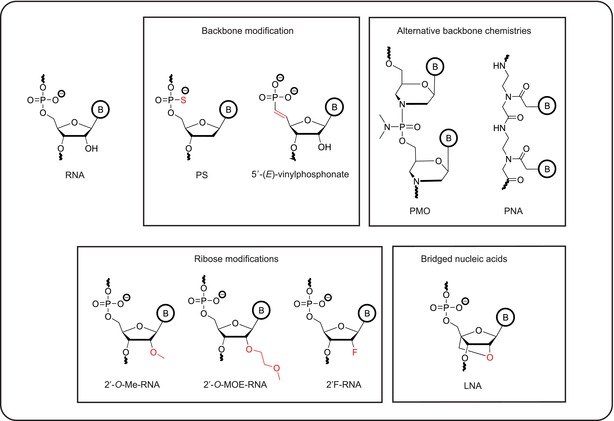

**Figure 2:**
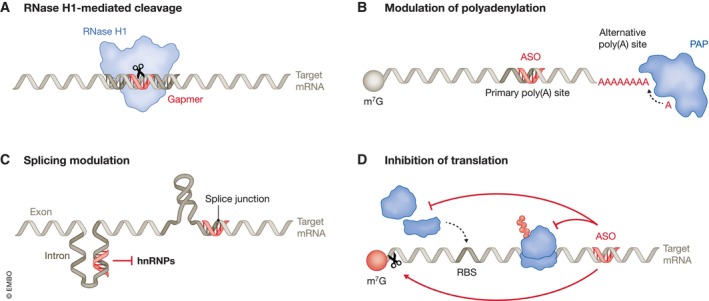
Molecular mechanisms of ASOs ASOs are extensively modified RNA analogs that bind to complementary sequences in target RNAs. They act via various molecular mechanisms such as (A) RNase H1‐mediated RNA cleavage induced by gapmers, which have a central core of deoxyribonucleotides (pink) flanked by 2′ modified nucleotides at both the 5′ and 3′ ends (gray), (B) modulation of polyadenylation, (C) modulation of splicing either by directly blocking splice junctions or by interfering with the binding of splice modulators, e.g heterogeneous nuclear ribonucleoproteins (hnRNPs) or (D) inhibition of translation by blocking ribosome scanning, interfering with translation initiation factors or causing the cleavage of the mRNA 5′ cap structure (m^7^G).

ASOs alter mRNA expression through a variety of mechanisms that either trigger the decay of target RNAs or prevent processing steps such as RNA splicing or translation (Fig 2; Crooke *et al*, 2021a). One important mode of action is the induction of Ribonuclease H1 (RNase H1)‐mediated cleavage and subsequent degradation (Fig 2A). RNase H1 is an endonuclease that cuts RNA in double‐stranded RNA:DNA hybrids. The resulting RNA fragments are degraded by 5′‐ and 3′‐exonucleases. RNase H1 requires 8–10 contiguous ribonucleotide‐containing base pairs (bp) in a substrate for optimal activity. This has led to the conception of “gapmers” — ASOs that have a central core of deoxyribonucleotides to support RNase H1‐mediated cleavage, which is flanked by 2′ modified nucleotides at both the 5′ and 3′ ends. This design enhances the affinity of the ASO for its target RNA and increases its resistance to nucleases (Crooke *et al*, 2021a).

In addition to RNase H1‐induced degradation, there are other mechanisms by which ASOs can alter mRNA levels. For example, they can redirect mRNA polyadenylation and thereby modify RNA stability (Vickers *et al*, 2001; Fig 2B). ASOs can also act on precursor mRNA to modulate splicing, either by directly blocking splice junctions or by interfering with the binding of splice modulators, proteins that promote or inhibit splicing (Hodges & Crooke, 1995; Hua *et al*, 2008; Rigo *et al*, 2012a; Fig 2C). These effects can be the goal itself, as in diseases caused by aberrant splicing (see sections on spinal muscular atrophy (SMA) or Duchenne muscular dystrophy (DMD) below). Alternatively, changes in splicing patterns may lead to mRNAs containing premature termination codons, which are selectively degraded through nonsense‐mediated mRNA decay (Ward *et al*, 2014). Moreover, ASOs can directly interfere with mRNA translation (Fig 2D). This can trigger no‐go decay (Liang *et al*, 2019), an mRNA quality control mechanism in which mRNAs with stacks of stalled ribosomes are recognized and degraded. Alternatively, ASOs can be designed to inhibit translation initiation by blocking ribosome scanning or by interfering with the interaction between mRNA and translation initiation factors (Melton, 1985; Baker *et al*, 1997). Additionally, ASOs can trigger the cleavage of 5′ cap structures, which inhibits translation and leads to mRNA decay (Baker *et al*, 1999; Fig 2D).

ASO delivery in a clinical setting faces challenges, including their degradation by serum nucleases, renal clearance, poor tissue penetration, and inefficient cellular uptake. The chemical modifications of ASOs discussed above and in Box 2 help alleviate some of these problems (Roberts *et al*, 2020). For example, the commonly used phosphorothioate backbone promotes interactions with serum proteins, which slows excretion by the kidneys (Sands *et al*, 1994). Chemical modifications also promote cellular uptake by cell surface receptors that facilitate oligonucleotide endocytosis (Koller *et al*, 2011; Roberts *et al*, 2020). Once inside the cell, ASOs must escape the endosome in order to be active, posing an additional challenge (Dowdy, 2023).

**Figure 3:**
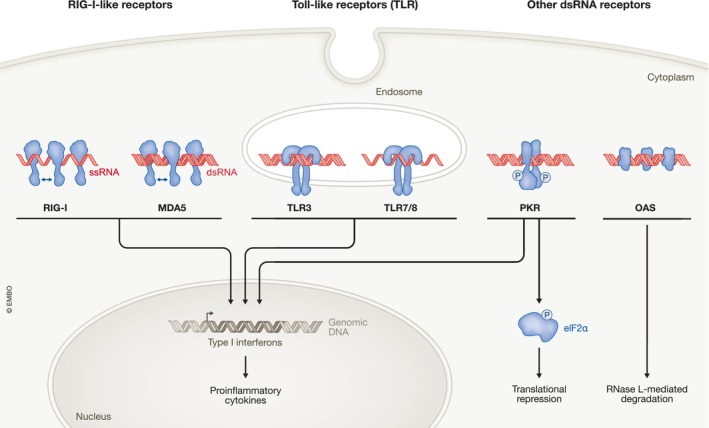
Cellular response to exogenous RNA Exogenous RNA is recognized by various innate immune receptors such as retinoic‐acid‐inducible gene I (RIG‐I), melanoma differentiation‐associated protein 5 (MDA5), endosome associated Toll‐like receptors (TLRs), protein kinase RNA‐activated (PKR) and 2′–5′ oligoadenylate synthase (OAS). Upon detection of their nucleic acid activator, these sensors initiate innate immune responses that often result in the production of Type I interferons, pro‐inflammatory cytokines, translational repression via phosphorylation of eukaryotic translation initiation factor 2 α (eIF2α) and RNA degradation by ribonuclease L (RNase L).

Another obstacle for ASO therapeutics is their immunogenicity. The human immune system recognizes both single‐stranded and double‐stranded RNA via pattern recognition receptors (PRRs; Takeuchi & Akira, 2010; Okude *et al*, 2020; Fig 3). Extracellular recognition is mediated by endosomal Toll‐like receptors (TLRs), especially TLR‐3, −7 and −8 (Lind *et al*, 2022). Intracellular recognition occurs via cytoplasmic defense mechanisms such as RIG‐I‐like receptors, protein kinase R (PKR), and oligoadenylate synthases (OASes; Hur, 2019). Activation of these pathways can trigger inflammatory responses, arrest of cellular translation, and RNA degradation, respectively. Therefore, 2′‐ribose modifications are commonly applied to synthetic RNA therapeutics to reduce their immunogenicity (Box 2; Crooke *et al*, 2021a).

Progress in understanding the molecular mechanisms of activity, distribution, cellular uptake, and toxicity of ASOs provides a framework for adapting this versatile technology to many diseases. While all currently approved ASO drugs are for use in patients with rare diseases (Table 1), many ASOs in clinical development are intended to treat common diseases such as cardiovascular and metabolic diseases and cancer. Although widespread implementation still faces challenges such as cell‐type‐specific delivery and potential adverse effects upon chronic treatment, ASO therapies are expected to have a substantial impact on many diseases that currently have limited or no treatment options.

**Table 1:** FDA‐approved RNA therapeutics.

Product	Target	Mechanism of action	Indication	Route of delivery	Company	Approval year
ASOs						
Formivirsen	CMV mRNA	Downregulation	CMV retinitis	IVT	Ionis Pharmaceuticals, Novartis	1998 (withdrawn 2002)
Mipomersen	Apolipoprotein B‐100 mRNA	Downregulation	Familial Hypercholesterolemia	SC	Ionis Pharmaceuticals	2013
Nusinersen	SMN2 pre‐mRNA	Splicing modulation	Spinal muscular atrophy	ITH	Ionis Pharmaceuticals, Biogen	2016
Eteplirsen	Exon 51 of dystrophin pre‐mRNA	Splicing modulation	DMD	IV	Sarepta Therapeutics	2016
Inotersen	TTR mRNA	Downregulation	Transthyretin‐mediated amyloidosis	SC	Ionis Pharmaceuticals	2018
Golodirsen	Exon 53 of *DMD*	Splicing modulation	DMD	IV	Sarepta Therapeutics	2019
Volanesoren	Apolipoprotein CIII mRNA	Downregulation	Familial chylomicronemia syndrome	SC	Ionis Pharmaceuticals, Akcea	2019
Viltolarsen	Exon 53 of dystrophin pre‐mRNA	Splicing modulation	DMD	IV	NS Pharma, Inc	2020
Casimersen	Exon 45 of dystrophin pre‐mRNA	Splicing modulation	DMD	IV	Sarepta Therapeutics	2021
RNAi‐based therapeutics						
Patisiran	TTR mRNA	Downregulation	Transthyretin‐mediated amyloidosis	IV	Alnylam	2018
Givosiran	ALS1 mRNA	Downregulation	Acute hepatic porphyria	SC	Alnylam	2020
Lumasiran	HAO1 mRNA	Downregulation	Primary hyperoxaluria type 1	SC	Alynlam	2020
Inclisiran	PCSK9	Downregulation	Atherosclerotic cardiovascular disease	SC	Novartis	2021
Vutrisiran	TTR mRNA	Downregulation	Transthyretin‐mediated amyloidosis	SC	Alynlam	2022
mRNA therapeutics						
BNT162b2	SARS‐CoV‐2 Spike mRNA	Expression of SARS‐CoV‐2 Spike protein	COVID‐19	IM	BioNTech, Pfizer	2020
mRNA‐1273	SARS‐CoV‐2 Spike mRNA	Expression of SARS‐CoV‐2 Spike protein	COVID‐19	IM	Moderna	2020

IM, intramuscular; ITH, intrathecal; IV, intravenous; IVT, intravitreal; SC, subcutaneous.

### RNAi‐based therapeutics

**Figure 4:**
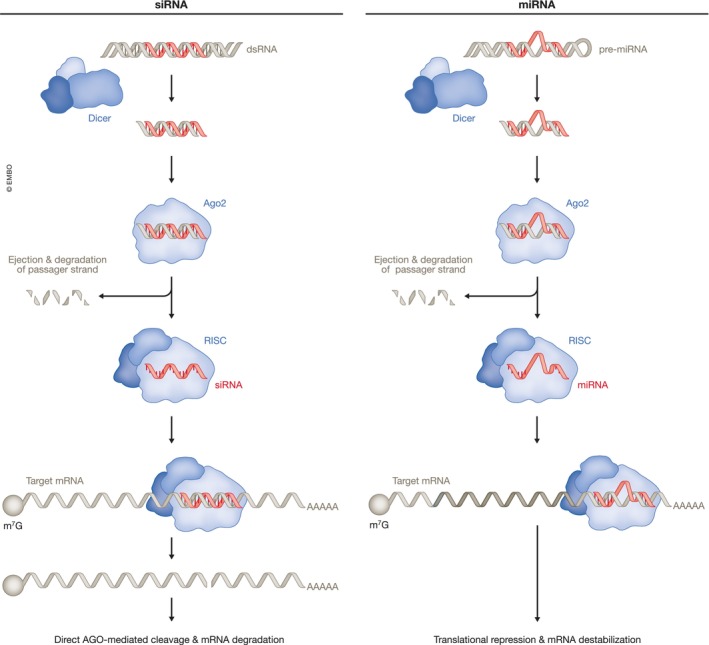
RNA interference RNA interference (RNAi) pathways are guided by small interfering RNAs (siRNAs; left) or microRNAs (miRNAs; right). siRNAs typically derive from exogenous sources while canonical miRNA precursors are derived from transcripts with internal hairpins. After endonucleolytic processing by Dicer, the RNA is loaded into Ago2 forming the RNAi‐induced silencing complex (RISC). siRNAs recognize targets with perfect or near‐perfect sequence complementarity and direct mRNA cleavage by Ago2. miRNAs typically recognizes target sites with imperfect complementarity, usually in the 3′ untranslated region (3′ UTR) of mRNAs and induce silencing by translational repression and target deadenylation and destabilization. miRNAs with perfect complementarity can also induce endonucleolytic cleavage by Ago2.

In 1998, Andrew Fire and Craig Mello identified double‐stranded RNA as the trigger for RNA interference in *Caenorhabditis elegans* (Fire *et al*, 1998; Box 1). Their observations challenged the prevailing view that antisense RNAs acted by directly binding and sterically interfering with target mRNA expression, and led to the discovery of an enzymatic silencing mechanism. Double‐stranded RNAs, either supplied exogenously or expressed within cells as precursor RNAs with stem loops or short hairpin structures, are converted by the cytoplasmic RNase III enzyme Dicer to small interfering RNAs (siRNAs) or microRNAs (miRNAs), respectively. Target mRNA regulation is mediated by the RNA‐induced silencing complex (RISC), a ribonucleoprotein complex composed of the siRNA or miRNA, which acts as the specificity determinant, and an Argonaute protein, which, together with other complex components, acts as the effector molecule (Meister, 2013; Ipsaro & Joshua‐Tor, 2015). siRNAs interact with their targets with perfect or near‐perfect complementarity and induce sequence‐specific cleavage of mRNAs through the slicer activity of Argonaute 2 (Ago2; Zamore *et al*, 2000; Liu *et al*, 2004). In contrast, miRNAs tend to interact with imperfectly complementary targets and cause translational repression and transcript degradation (Pillai *et al*, 2005; Wu *et al*, 2006; Djuranovic *et al*, 2012; Fig 4). The RNAi pathway in human cells is remarkably efficient due to the ability of the activated RISC to direct multiple rounds of RNA cleavage (Hutvágner & Zamore, 2002). RNAi‐based therapies take advantage of this feature and of the versatility and programmability of the RNAi machinery, but the need to engage this machinery also creates constraints on the design of siRNA drugs.

Generally, synthetic RNAi triggers are perfectly base‐paired double‐stranded RNAs that must be unwound so that one of the strands can be selected as the “guide” strand to be loaded into RISC. Since only the antisense strand hybridizes to the target mRNA, RNAi drug design must ensure that the correct strand is chosen. For example, siRNAs with a blunt end on one side and a 2‐nt 3′ overhang on the other tend to bias guide strand selection to the strand with the 3′ overhang (Sano *et al*, 2008). In addition, chemical modifications can also promote antisense strand loading (Varley & Desaulniers, 2021). RNAi triggers that are longer than 21 bp require Dicer for cleavage and handoff to RISC. Notably, Dicer processing is linked to a more reliable selection of the antisense strand as the RISC guide (Snead *et al*, 2013). Shorter siRNAs bypass the early steps of the RNAi pathway and can be loaded into RISC directly. This has the advantage that they are less likely to interfere with gene regulation by endogenous miRNAs (Grimm *et al*, 2006).

siRNA drugs face similar challenges as ASOs in terms of delivery, stability, and immunogenicity. Fortunately, the insights into backbone, base, and sugar modifications initially established for ASO therapeutics (Box 2) largely apply to siRNA therapeutics, although the molecular requirements for effective recruitment of the RNAi machinery impose limitations on the chemical modification of siRNAs (Khvorova & Watts, 2017). Moreover, the delivery of duplex siRNAs is more challenging than the delivery of single‐stranded ASOs, partly due to their increased size and hydrophilicity. In siRNAs, the external‐facing phosphate groups create a hydrated surface that adheres poorly to cells leading to their rapid excretion from the body. As a result, researchers have developed delivery vehicles such as lipid nanoparticles and targeting ligands (Box 3) to improve siRNA delivery. Further, the use of chemical optimization strategies is leading to the design of modified siRNAs that will expand the reach of RNAi therapeutics (Davis *et al*, 2022).

Box 3 **RNA delivery**

RNA is large and negatively charged, making it difficult to cross cell membranes. It is also rapidly degraded by nucleases that are active in all fluids of the body. Therefore, several approaches have been developed to increase the efficiency of *in vivo* delivery of RNA therapeutics, including encapsulation into nanoparticles. Lipid‐based nanoparticles (LNPs) are the most advanced for mRNA delivery and offer benefits such as ease of formulation, biocompatibility, and a large carrying capacity (Chaudhary *et al*, 2021; Hou *et al*, 2021). LNPs typically include ionizable lipids, cholesterol, a phospholipid, and a PEGylated lipid. Initially, cationic lipids were used due to their positive charge, which facilitated the encapsulation of negatively charged RNA (Kauffman *et al*, 2016). However, cationic lipids trigger toxic and proinflammatory responses (Cui *et al*, 2018). To overcome these drawbacks, ionizable lipids were developed (Cullis & Hope, 2017). These lipids are neutral at physiological pH, improving their safety and extending their circulation time. In acidic conditions, they are positively charged. This aids RNA packaging, but also promotes fusion with the endosomal membrane after cellular uptake to release the cargo into the cytoplasm. The other lipid components of LNPs play a role in their formation and function. Cholesterol enhances stability and aids membrane fusion during uptake, helper lipids modulate nanoparticle fluidity and enhance efficacy, and PEGylated lipids stabilize LNPs and regulate their size and half‐life (Chaudhary *et al*, 2021). Polymers, which offer similar advantages to lipids, and cell‐penetrating peptides can also deliver mRNA into cells. Chemical conjugation to trivalent N‐acetylgalactosamine (GalNAc; Nair *et al*, 2014; Matsuda *et al*, 2015; Rajeev *et al*, 2015), which is recognized by the hepatocyte‐restricted asialoglycoprotein receptor (ASGPR) represents an efficient way of increasing liver uptake, especially of short RNAs.

The first therapy based on RNAi‐mediated gene silencing received FDA approval in 2018. Patisiran is a double‐stranded siRNA indicated for the treatment of hereditary transthyretin‐mediated amyloidosis (hATTR; Adams *et al*, 2018). This is a progressive neurodegenerative disease caused by the deposition of amyloid fibrils formed by the misfolded protein transthyretin. Patisiran silences transthyretin mRNA in the liver and decreases serum levels of the protein, thus reducing the amyloid deposits. It is composed of two modified 21‐mer oligonucleotides and encapsulated in a lipid nanoparticle formulated for hepatocyte uptake. Vutrisiran, a successor to patisiran, came on the market in 2022. It uses the same RNAi mechanism but takes advantage of enhanced stabilization chemistry. This siRNA is coupled to N‐acetylgalactosamine (GalNAc; Box 3), which increases its uptake in liver cells and allows the administration of lower doses (Adams *et al*, 2023). While patisiran required intravenous injection every 3 weeks, treatment with vutrisiran involves only one subcutaneous injection every 3 months. Currently, five RNAi‐based drugs have been approved by the FDA (Table 1), and the many oligonucleotide drugs currently in pre‐clinical and clinical development indicate that RNAi therapeutics will soon be used in a broad range of applications. For example, several strategies to achieve extrahepatic delivery are being explored, including antibody conjugates (Cuellar *et al*, 2015; Dugal‐Tessier *et al*, 2021; Malecova *et al*, 2023), peptide conjugates (Klabenkova *et al*, 2021), hydrophobic (Biscans *et al*, 2019) or lipophilic conjugates (Brown *et al*, 2022) as well as multivalency (Alterman *et al*, 2019). In addition, programmable siRNA pro‐drugs that are activated in response to specific cellular RNA biomarkers show promise in selective targeting of diseased cells over healthy bystander tissue (Han *et al*, 2022).

miRNAs are important gene regulators that influence many physiological processes linked to disease, making them attractive therapeutic targets in their own right (Rupaimoole & Slack, 2017). miRNAs require only partial complementarity for target recognition. Therefore, a single miRNA can interact with multiple mRNAs with different affinities. Interfering with or mimicking miRNA function thus enables the simultaneous manipulation of complex gene expression networks. Targeting multiple, potentially compensatory pathways at once is appealing, but this approach also carries the risk of unforeseen side effects.

miRNA therapeutics come in two flavors: miRNA mimics and antimiRs. miRNA mimics are synthetic oligonucleotide duplexes that imitate the function of a naturally occurring miRNA akin to siRNA drugs. antimiRs are structurally similar to ASOs and are designed to bind directly to the mature strand of the targeted miRNA and to block its function. More details on this topic can be found in a comprehensive review on miRNA‐targeted therapeutics (Rupaimoole & Slack, 2017). For the purpose of this overview, we want to highlight the antimiR miravirsen as a notable example. Miravirsen targets miR‐122, an abundant liver miRNA that regulates lipid metabolism. miR‐122 also plays a critical role during infection with Hepatitis C virus (HCV), a major cause of chronic liver diseases such as cirrhosis and hepatocellular carcinoma. Binding of miR‐122 to the 5′‐untranslated region (UTR) of HCV RNA is essential for viral replication (Jopling *et al*, 2005), and miravirsen was designed to interfere with this process. It is composed of locked nucleic acid (LNA) ribonucleotides interspersed throughout a DNA phosphorothioate sequence that is complementary to miR‐122. Miravirsen hybridizes to mature miR‐122 and blocks its interaction with HCV RNA, inhibiting HCV replication (Lanford *et al*, 2010). It showed prolonged antiviral activity in initial clinical trials and was well‐tolerated in patients infected with HCV (Janssen *et al*, 2013), but the success of potent small‐molecule antiviral treatments for hepatitis C diminished the clinical need for miravirsen and its clinical development was recently discontinued.

### mRNA‐based therapeutics

The RNA drugs discussed above act as effectors, but RNA, in the form of mRNA, is also a carrier of genetic information that can serve as a therapeutic agent by mediating protein expression. Applications for mRNA‐based therapeutics include vaccines against infectious diseases and cancer as well as protein replacement. Clinically applied synthetic mRNAs are usually *in vitro* transcribed (IVT) from a DNA plasmid using a bacteriophage RNA polymerase. They are structured similarly to cellular mRNA and include elements such as a 5′ cap, a 5′ UTR, an open reading frame (OTR), 3′ UTR, and poly(A) tail. These features are important for mRNA translation and stability and therefore affect efficacy of the mRNA drug. The synthesized mRNA is purified to remove contaminants, reactants, and incomplete transcripts, which is essential to reduce immune‐stimulatory effects (Karikó *et al*, 2011). To maximize translation, modified nucleosides like pseudouridine and N1‐methylpseudouridine are often incorporated into the mRNA molecule. The use of these modified nucleosides, particularly modified uridine, also prevents the recognition of the IVT mRNA by the innate immune system, thus allowing for higher dosing (Karikó *et al*, 2005).

Early efforts to use IVT mRNA for therapeutic applications laid the groundwork for the rapid development of highly efficient mRNA vaccines against SARS‐CoV‐2 (Chaudhary *et al*, 2021). By the end of 2019, several preclinical and clinical studies had established the potential of mRNA vaccines to protect against pathogens such as Zika virus, respiratory syncytial virus (RSV), Influenza A virus, and rabies virus, but it was expected that it would take another 5–6 years before an mRNA vaccine would be approved for clinical use (Chaudhary *et al*, 2021). The COVID‐19 pandemic accelerated this development, and the mRNA vaccines developed by BioNTech/Pfizer and Moderna received approval within a mere 10 months. Both vaccines are formulated with ionizable lipid nanoparticles (LNPs; Box 3) and deliver a nucleoside‐modified mRNA encoding the viral spike glycoprotein. They demonstrated more than 90% efficacy in clinical trials. Aside from transient local and systemic reactions, no safety concerns were identified (Polack *et al*, 2020; Baden *et al*, 2021), although potential long‐term effects must be further evaluated.

mRNA vaccines are usually administered as a single injection into the skin, muscle, or subcutaneous space, where they are taken up by immune or non‐immune cells and translated into antigens that stimulate an immune response. In contrast to plasmid DNA and viral DNA vectors, IVT mRNA does not need to enter the nucleus to be effective. The high sensitivity of the immune system enables the generation of strong immune responses even at low antigen levels, making high and sustained expression of the IVT mRNA unnecessary.

The mRNA platform has several benefits for pandemic vaccine production, notably a rapid development time and cost‐effective, scalable production, allowing a fast response in case a new pandemic virus emerges. Other advantages include flexibility in antigen design and the ability to deliver multiple antigens in a single formulation. These features can be exploited in the development of “universal” vaccines that provide broad protection against multiple viral strains. While regulatory and approval pathways for these vaccines still need to be fully established, we are likely to see the development of more mRNA vaccines for infectious diseases in the near future.

Another promising application of mRNA vaccines is the personalized treatment of cancer (Sahin & Türeci, 2018). In ongoing clinical studies, researchers are using mRNA vaccines to stimulate an immune response against specific cancer‐associated neoantigens (Kranz *et al*, 2016; Sahin *et al*, 2017; Palmer *et al*, 2022; Rojas *et al*, 2023), with early results suggesting that these therapies can yield clinical benefit (Dolgin, 2023). These vaccines are individualized for each patient based on RNA sequencing analysis of their tumor tissue, and are designed to instruct the patient's immune system to target and attack the cancer cells (Lang *et al*, 2022).

mRNA vaccines can also be used to treat autoimmune diseases by selectively dampening autoimmune responses without compromising normal immune function (Krienke *et al*, 2021). This is achieved through systemic delivery of mRNA encoding disease‐related autoantigens, designed to be taken up and presented by lymphoid antigen‐presenting cells with low‐level surface expression of co‐stimulatory molecules. This leads to peripheral tolerance through reduction of effector T cells and development of regulatory T cell populations that mediate bystander immunosuppression (Krienke *et al*, 2021).

Besides mRNA vaccines, an obvious use of therapeutic mRNA is the expression of proteins that are absent or not functional in the body. This approach has the potential to treat a wide range of diseases, but comes with additional challenges like the need for high and sustained expression of the therapeutic protein in specific cell types, as well as difficulties in delivering mRNA to solid organs other than the liver. Treatment of chronic diseases would require repeated dosing, which can activate the immune system and reduce the effectiveness of the therapeutic protein, even with the use of modified mRNA and advanced delivery vehicles. The therapeutic protein itself can also elicit an immune response if it is not expressed endogenously in the body. To date, only a small number of clinical studies have shown promising results for this approach in terms of safety and efficacy. One notable example is the use of VEGF mRNA to promote vasculogenesis during cardiac regeneration (Anttila *et al*, 2020).

### Therapeutic genome editing

While mRNA‐based protein replacement therapies allow temporary expression of a functional copy of a transcript, therapeutic genome editing offers the promise of a permanent cure by correcting pathogenic mutations in genomic DNA. The clinical feasibility of this approach is the result of the discovery of ancient bacterial adaptive immune systems – the CRISPR‐Cas nucleases (Mojica *et al*, 2005; Barrangou *et al*, 2007; Brouns *et al*, 2008; Box 1) – and their development as genome editing tools.

**Figure 5:**
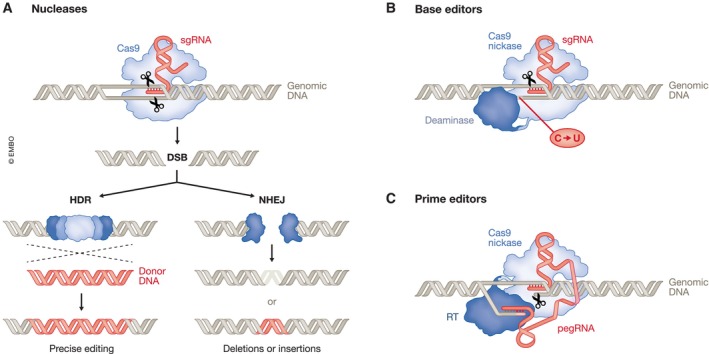
CRISPR‐Cas‐based genome editing tools (A) Cas9 nucleases are guided to their target region by a small guide RNA (sgRNA). There they generate double‐strand breaks (DSBs) that are repaired by either homology‐directed repair (HDR) or non‐homologous end joining (NHEJ). HDR repairs DSBs by homologous recombination using a donor DNA template. This can be the sister chromatid when it is present during the late S or G2 phase of the cell cycle or an exogenously supplied donor that supports gene correction. In contrast, NHEJ, the dominant path for DSB repair, often leads to uncontrolled nucleotide deletions or insertions. (B) Base editors mediate targeted single‐nucleotide conversions using a fusion between a Cas9 nickase and a deaminase domain, which modifies single bases through deamination. Nicking of the nondeaminated strand biases cellular DNA repair to replace the unedited strand, thereby resolving the mismatch and leading to stable conversion. Base editors are able to mediate targeted C > U, A > G, C > G, A > I and C > T conversions. (C) Prime editors consists of a reverse transcriptase (RT) fused to a Cas9 nickase. The Cas9 nickase binds to and nicks the non‐target DNA strand and the reverse transcriptase subsequently uses the resulting free 3′ end to copy the sequence of the prime‐editing guide RNA (pegRNA).

The first described and still widely used genome editing tool is the type‐II CRISPR‐Cas9 system from *Streptococcus pyogenes* (SpCas9; Deltcheva *et al*, 2011; Jinek *et al*, 2012). SpCas9 is an RNA‐guided DNA endonuclease that generates targeted double‐strand breaks (DSBs) at specific genomic loci (Gasiunas *et al*, 2012; Jinek *et al*, 2012). The enzyme recognizes its target sites through a small guide RNA that hybridizes to complementary regions in DNA (Fig 5A). This programmable RNA can be designed to guide Cas9 to genomic regions of interest (Cong *et al*, 2013; Jinek *et al*, 2013; Mali *et al*, 2013). The only sequence requirement is that the target sequence must be flanked on the 3′ side by a short protospacer adjacent motif (PAM). This prerequisite limits the genomic sites that can be edited to locations of PAM sequences. Cas nucleases isolated from different bacterial species recognize different PAM sequences, which broadens the targeting space. In addition to natural Cas variants, there are now lab‐evolved mutants with alternative PAM recognition (Hu *et al*, 2018; Nishimasu *et al*, 2018; Miller *et al*, 2020; Walton *et al*, 2020).

Once the Cas nuclease has been guided to a target site in the genome, it generates a DSB, which is subsequently repaired by cellular DNA repair systems, either by homology‐directed repair (HDR) or, more commonly, by error‐prone non‐homologous end joining (NHEJ) or microhomology‐mediated end joining (Fig 5A; Hustedt & Durocher, 2016). These repair pathways pose challenges for Cas nuclease‐mediated therapeutic genome editing. Specifically, although HDR in principle allows the precise correction of a mutation through the use of a donor DNA template to repair the break, this pathway is restricted to the S and G2 phase of the cell cycle, making it unusable for treating diseases that involve non‐dividing cells. And while the NHEJ pathway is active in non‐dividing cells, it ligates the broken DNA ends in a template‐independent manner. This results in small insertions and deletions at the break point, excluding the generation of exact site‐specific mutations. DSBs in genomic DNA can also cause large deletions, chromosomal translocations, or other chromosomal abnormalities (Giannoukos *et al*, 2018; Kosicki *et al*, 2018; Turchiano *et al*, 2021). While rare, this poses safety risks.

Recently developed precision genome editing strategies that do not rely on DSBs overcome these limitations. Prominent among these are base editors (BEs), which enable single‐nucleotide conversions in genomic DNA (Rees & Liu, 2018; Fig 5B). BEs are fusions of a Cas9 nickase (nCas9), that is a Cas9 variant that produces a single‐stranded rather than a double‐stranded break, and an enzyme that catalyzes a nucleobase deamination (Komor *et al*, 2016; Nishida *et al*, 2016). Like Cas9 nucleases, the nCas9‐deaminase fusion is directed to a genomic target site by a small guide RNA. Base pairing between the guide RNA and the target DNA exposes a region of single‐stranded DNA that is then accessible to deamination. The resulting base mismatch is resolved through cellular repair mechanisms. While the current BE toolbox mediates a range of single nucleotide conversions, base editing does not yet extend to all possible exchanges, and it cannot perform targeted insertions or deletions. Nevertheless, BEs show promising results in pre‐clinical studies (Arbab *et al*, 2023; Mayuranathan *et al*, 2023) and are progressing towards the clinic, for example, in an early‐stage clinical trial for the treatment of familial hypercholesterolemia (Kingwell, 2022).

Prime editors (PEs) provide additional flexibility to engineer changes beyond single‐nucleotide conversions (Chen & Liu, 2023; Fig 5C). PEs consist of a Cas9 nickase fused to a reverse transcriptase (Anzalone *et al*, 2019). They use an engineered prime editing guide RNA (pegRNA) that directs the enzyme to a specific target locus, and also serves as a template for the edit of interest. PEs first nick the non‐target DNA strand and use the resulting free 3′ end to prime reverse transcription using the pegRNA as a template. While several examples of *in vivo* gene editing using PEs have been reported (Newby & Liu, 2021), their efficiency currently lags behind BEs (Wang & Doudna, 2023). Still, recent improvements bode well for their future (Chen *et al*, 2021; Nelson *et al*, 2022).

For therapeutic applications, delivery remains a major bottleneck. Current delivery strategies are divided into two types of approaches: *ex vivo*, where cells are removed from the patient, edited outside the body, and reintroduced into the patient; and *in vivo*, where cells are edited directly in the patient following delivery of CRISPR components. *Ex vivo* approaches are often used for editing hematopoietic stem and progenitor cells, but most other cell types are not amenable to *ex vivo* manipulation and transplantation. *In vivo* approaches offer treatment for a wider range of genetic diseases, but require efficient and safe delivery of the editing agents to specific cell types.

Common delivery vehicles include viral vectors, typically adeno‐associated virus (AAV), adenoviruses or lentiviruses, as well as nanoparticles (Raguram *et al*, 2022; Box 3). Viral delivery offers advantages in terms of efficiency and tissue selectivity. AAVs are especially attractive because of their inherent tissue tropism and clinically manageable immunogenicity, but they have a low packaging capacity. Adeno‐ and lentiviruses offer higher packaging efficiencies, but have immunogenicity concerns. Production at scale and a good manufacturing practice at affordable cost are other unresolved issues for viral‐based delivery. Nanoparticles, on the other hand, are easier to produce and are often considered safer. Yet, they have lower delivery efficiency compared to viral vectors, and when systemically delivered naturally accumulate in the liver. Recently, virus‐like particles (VLPs) have emerged as promising delivery platforms. These viral protein assemblies can package desired cargo and transduce cells, but mostly lack viral genetic material (Raguram *et al*, 2022). They offer the high delivery efficiencies of viral vectors without the associated safety concerns and have the potential to be targeted to specific cell types by exploiting the cellular tropism of different viral envelope glycoproteins (Banskota *et al*, 2022). Nevertheless, the feasibility of scaling up production of VLPs in quantities required for pre‐clinical or clinical studies still needs to be established.

Immunogenicity concerns associated with the *in vivo* delivery of gene editing agents also need to be considered. These include potential preexisting immunity to delivery vehicles (Weber, 2021) or cellular immunity to Cas9 and other components of the gene editing machinery (Charlesworth *et al*, 2019; Wagner *et al*, 2021a). In addition, their prolonged expression in edited cells might provoke adaptive immune responses and may increase the chances of off‐target activity. Therefore, transient delivery that minimizes exposure to gene editing agents is desirable.

Therapeutic approaches that use genome editing to treat hereditary diseases are currently mostly in the preclinical stage, although some have advanced to clinical trials. For example, Cas nuclease‐mediated gene therapy has achieved promising initial clinical results in the treatment of beta‐thalassemia and sickle cell disease, two inherited blood disorders caused by reduced production of hemoglobin. The approach taken in the most advanced trial uses *ex vivo* genome editing and aims to reactivate the synthesis of fetal hemoglobin, which is normally deactivated shortly after birth (Frangoul *et al*, 2021). This strategy offers treatment to both diseases and circumvents the need to precision edit a disease‐associated mutation. Instead, it seeks to disrupt an erythroid‐specific enhancer region of BCL11A, a transcription factor that represses expression of fetal hemoglobin (Canver *et al*, 2015; Wu *et al*, 2019).

Notable examples for successful *in vivo* delivery of CRISPR‐based therapeutics are clinical trials to treat the neurodegenerative disease hATTR (Gillmore *et al*, 2021) and the treatment of Leber congenital amaurosis 10 (LCA10), a type of congenital blindness (Maeder *et al*, 2019). The former constitutes the first systemic *in vivo* delivery of CRISPR components to the human liver using targeted LNPs, while the latter involves direct injection of AAV harboring Cas9 and two small guide RNAs into the eye.

## Opportunities and challenges for RNA therapeutics exemplified by specific genetic diseases

Some RNA‐based therapies have shown remarkable clinical success while others have unexpectedly encountered limited efficacy. The recent suspension of the CRISPR‐editing approach to LCA10, the rare inherited blindness disorder mentioned above, is a case in point. The setting could be considered a best‐case‐scenario because the eye is easily accessible for direct injection of therapeutic modalities and it has an immune‐privileged status (Suh *et al*, 2022). The strategy – CRISPR‐Cas‐mediated correction of a splice defect in the *CEP290* gene, which causes LCA10 – had shown promising preclinical results (Maeder *et al*, 2019). Nevertheless, although the treatment was well tolerated, only three out of 14 patients experienced clinically meaningful vision improvement, based on company announcements. These disappointing results underline that the road ahead is not without challenges. In this section, we will discuss three genetic disorders that have been targeted by RNA‐based therapeutics, with varying success. We will consider the specific challenges associated with each of these diseases, and highlight how RNA drugs could facilitate treatment.

### Spinal muscular atrophy

**Figure 6:**
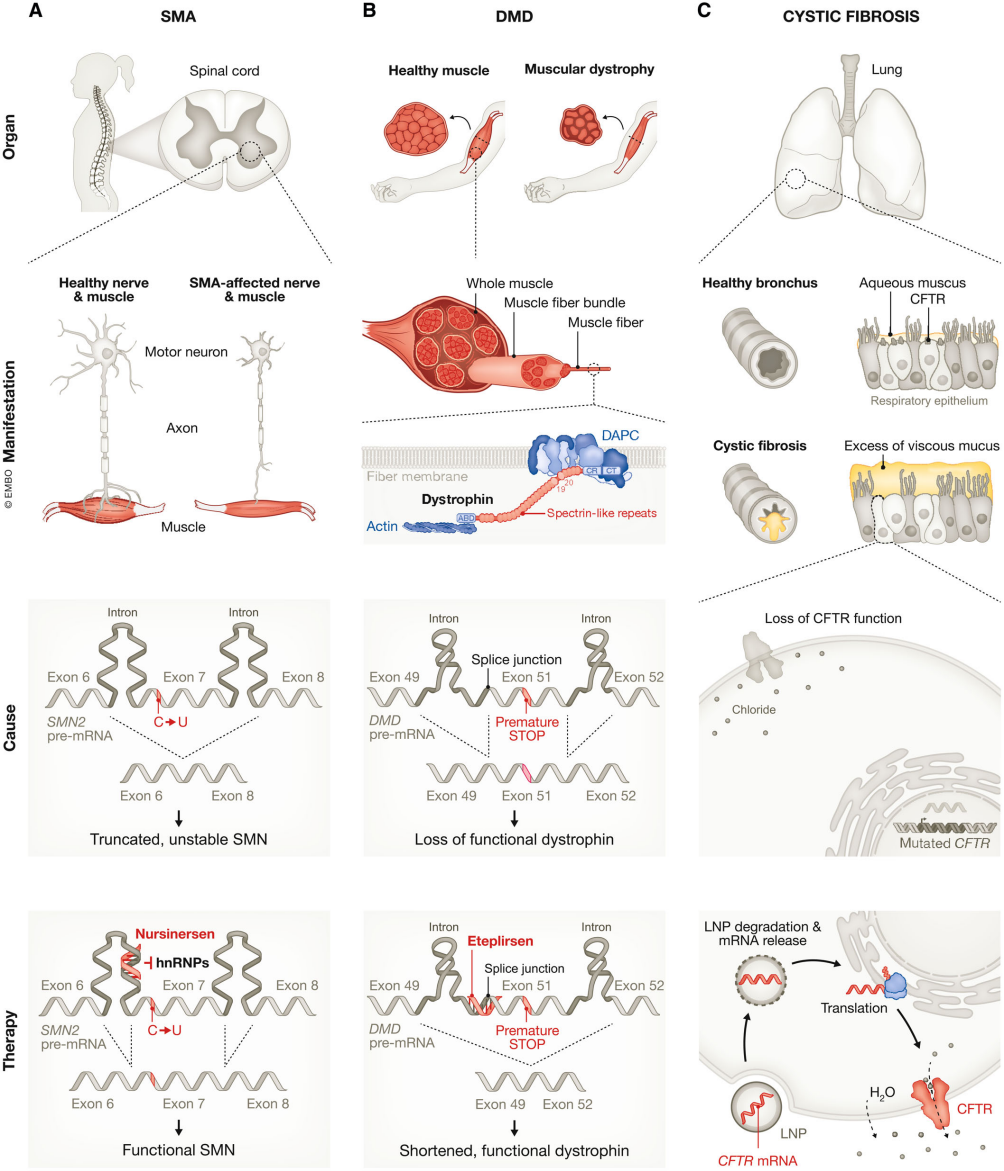
Disease mechanisms and RNA‐based therapeutic approaches for SMA, DMD and cystic fibrosis (A) SMA results in the loss of motor neurons in the spinal cord leading to muscular atrophy. The disease is caused by a lack of functional SMN protein due to mutations in the *SMN1* gene and aberrant splicing of its paralog *SMN2*. The *SMN2* splicing defect is due to a synonymous C‐to‐T substitution within exon 7, which causes the production of a truncated, unstable polypeptide. Binding of the ASO nusinersen to the SMN2 pre‐mRNA displaces the splice repressor hnRNP, resulting in the production of a mature mRNA that includes exon 7 and translation of the full‐length SMN protein. (B) DMD is characterized by progressive muscle degeneration due to mutations in the protein dystrophin. Dystrophin has a modular organization with an N‐terminal actin binding domain (ABD), a rod domain consisting of 24 spectrin‐like repeats and four interspersed hinges, a cysteine‐rich domain (CR) and a C‐terminal (CT). Dystrophin interacts with the actin cytoskeleton and the dystrophin‐associated protein complex (DAPC), a transmembrane scaffold, thereby supporting the maintenance of muscle cell integrity and contractility. One of the more common causes of the disease is a change in the *DMD* reading frame caused by deletion of exon 50, which leads to a nonsense mutation and the loss of functional dystrophin. Eteplirsen binds to exon 51 and favors exclusion of exon 51, thereby restoring the reading frame and enabling production of partially functional, internally deleted dystrophin. (C) Cystic fibrosis leads to the accumulation of excessively viscous mucus that obstruct passageways, e.g. in the lung. The disease is caused by dysfunction of the cystic fibrosis transmembrane conductance regulator (CFTR), an anion channel that maintains fluid‐ and electrolyte‐homeostasis. One of the RNA‐based therapeutic approaches currently in development is the delivery of CFTR mRNA packaged into lipid nanoparticles (LNPs) to drive cellular expression of functional CFTR.

One of the key success stories of ASO therapeutics is nusinersen, approved for the treatment of spinal muscular atrophy (SMA). SMA is an autosomal recessive neuromuscular disease caused by deletions or loss‐of‐function mutations in the gene *survival motor neuron 1 (SMN1)* (Lefebvre *et al*, 1995). Without functional SMN protein, the motor neurons in the spinal cord and brain stem degenerate, resulting in muscle weakness and atrophy (Fig 6A). Of the infants born with the most severe form of SMA, 60% show symptoms before 6 months of age and the median life expectancy is less than 2 years. Until nusinersen came on the market in 2016, there were no approved therapies for SMA, and medical care focused on supportive and palliative measures.

Humans have a second gene, *survival motor neuron 2 (SMN2)* that encodes an identical SMN protein. But *SMN2* contains a synonymous C‐to‐T substitution within exon 7 that weakens the binding of splice activators to the SMN2 pre‐mRNA (Hofmann *et al*, 2000; Cartegni & Krainer, 2002). This leads to aberrant splicing, with 90% of mature SMN2 transcripts lacking exon 7 and producing a truncated, unstable polypeptide (Fig 6A). Instead of targeting *SMN1*, the tactic chosen to restore functional levels of SMN protein was to use ASOs to promote *SMN2* exon 7 inclusion (Rigo *et al*, 2012b). An early approach was to engineer bifunctional ASOs that operated as synthetic splice activators: a peptide mimicking a splice activator was covalently linked to an ASO that hybridized to exon 7 (Cartegni & Krainer, 2003). Over the years, the strategy to control exon 7 inclusion was optimized. It was shown that ASOs targeting a site near the 5′ splice site in SMN2 intron 7 could efficiently promote exon 7 inclusion without the need of an appended peptide moiety. They acted by preventing binding of the splice repressors hnRNP A1 and hnRNP A2 (Hua *et al*, 2008; Rigo *et al*, 2012a; Fig 6A). In addition, chemical modifications in the backbone and nucleotides of the ASOs improved their pharmacological properties.

Based on preclinical studies in mice and non‐human primates (Hua *et al*, 2011; Passini *et al*, 2011; Rigo *et al*, 2014), nusinersen advanced to clinical development and underwent highly successful clinical trials (Finkel *et al*, 2016, 2017). An interim analysis of a phase III clinical trial for patients with infantile‐onset SMA showed that 40% of children treated with nusinersen achieved improvement in motor functions, such as head control, sitting, rolling, crawling, standing and walking, whereas none of the control patients did. These results led to the early termination of the trial and the drug was approved for use in the US in 2016. It has since become available in over 40 countries. Nusinersen has revolutionized the treatment of SMA patients, and it is also the first antisense drug to achieve a substantial commercial success. This feat, along with the approval of seven other ASOs (Table 1) for the treatment of rare genetic diseases, suggests that the ASO technology has potential to fulfill the hopes placed in it.

### Duchenne muscular dystrophy

Other monogenic diseases such as Duchenne muscular dystrophy (DMD) have proven more challenging to treat by RNA therapeutics. DMD is a progressive X‐linked muscle‐wasting disease caused by deletions, duplications, or point mutations in the *DMD* gene (Koenig *et al*, 1987). *DMD* is one of the largest genes in the human genome, spanning ~2.3 Mb of DNA, and exhibits a complex intron‐exon organization. Thousands of different *DMD* mutations have been found in patients with DMD. They cluster in hotspot regions and approximately 47% of patients carry mutations in exons 45–55 (Nakamura *et al*, 2017). *DMD* encodes the protein dystrophin (Hoffman *et al*, 1987), which protects muscle fibers from contraction‐induced injury (Fig 6B). Dystrophin has a modular organization with an N‐terminus that interacts with the actin cytoskeleton and a C‐terminus that anchors the protein to a transmembrane scaffolding complex, termed the dystrophin‐associated protein complex (DAPC; Gao & McNally, 2015). The central region of dystrophin, which is encoded by the genomic region that includes exons 45–55, is composed of a rod‐domain divided into 24 redundant spectrin‐like repeats and four interspersed hinges (Fig 6B). Dystrophin and the DAPC have both structural and signaling roles that support the maintenance of muscle cell integrity and contractility. DAPC disassembly caused by the absence of functional dystrophin has severe effects on muscle cell function. Patients experience increasing difficulties in movement and eventually need assisted ventilation; the disease inevitably culminates in premature death from respiratory and cardiac failure.

Therapeutic approaches attempting to maintain muscle cell function in DMD include treatment with agents that block inflammation, fibrosis, calcium overload, and oxidative stress (Duan *et al*, 2021). Yet none of these treatments address the primary cause of the disease – lack of functional dystrophin. Extensive efforts have been directed toward the development of ASOs that restore the reading frame of dystrophin transcripts. This strategy leads to the expression of a partially functional, internally deleted dystrophin with fewer spectrin‐like repeats, but both terminal interaction domains. This “exon‐skipping” approach is based on the observation that naturally occurring mutations that maintain the *DMD* reading frame cause Becker muscular dystrophy, a milder form of the disease with later onset and slower progression (Mercuri *et al*, 2019).

The first drug approved by the FDA as a specific DMD therapy was the ASO eteplirsen, which masks a splice acceptor sequence in exon 51 of *DMD* thereby promoting restoration of dystrophin expression in patients with deletions of exon 50 (Mendell *et al*, 2016; Alfano *et al*, 2019; Fig 6B). It was followed by ASOs designed to skip exon 53 (golodirsen (Frank *et al*, 2020) and viltolarsen (Roshmi & Yokota, 2019)) or exon 45 (casimersen (Wagner *et al*, 2021b)). FDA approval was based on low levels of dystrophin restoration in small cohorts of patients rather than on confirmation of functional efficacy and continued approval is contingent upon validation of a clinical benefit in on‐going confirmatory trials. Based on the initial clinical trial data, the European Medicines Agency (EMA) refused approval of eteplirsen, while approval of the other three ASOs is awaited. In the interim, efforts are underway to develop more effective ASO therapies. All four FDA‐approved ASOs are uncharged phosphorodiamidate morpholino oligomers (PMOs) and different chemical modifications and conjugation to arginine‐rich or muscle‐homing peptides are being explored to improve ASO efficacy and uptake by skeletal muscle and heart. Although the approved ASOs are generally well tolerated, the durability of the response is short. Because of protein and ASO turnover, the drugs need to be administered once weekly as a 35–60 min intravenous infusion.

CRISPR‐based gene editing of *DMD* would avoid life‐long treatment. Restoration of only a small amount of the normal level of dystrophin provides therapeutic benefits in mice (Long *et al*, 2014). Since skeletal muscles are multinucleated, this might be achieved if only a fraction of the myonuclei are corrected. On the other hand, muscle cells are postmitotic, so that precise *DMD* correction via HDR is not feasible. Instead, the strategy is reliant on error‐prone NHEJ and depends on the redundancy in dystrophin's central rod domain, which permits deletion of internal segments if the ORF is maintained. While proof of concept has been achieved confirming that genome editing can restore dystrophin levels in cells and animal models (Long *et al*, 2016; Min *et al*, 2019, 2020), challenges have to be overcome to apply this approach systematically in humans. These include optimal delivery of genome‐editing components, the risk of off‐target editing and a potential immune response against Cas9 (Olson, 2021).

“Classic” gene therapy approaches to restore dystrophin expression using viral vectors are in more advanced stages. While most viruses do not have a natural tropism for skeletal or heart muscle, AAVs can infect these tissues efficiently. However, due to AAV's limited carrying capacity, only micro‐dystrophin constructs that lack all but the most crucial domains can be used. Phase 1 clinical trials have shown micro‐dystrophin expression in muscle fibers but it is not yet established if treatment will ameliorate disease progression (Mendell *et al*, 2020). Despite this uncertainty, elevidys, a micro‐dystrophin AAV gene therapy has recently been granted accelerated approval by the FDA. Although it is unclear which therapeutic modality will ultimately prove to be effective, there is hope that continuous research efforts will result in the development of a successful DMD therapy.

### Cystic fibrosis

In the competitive drug development market, RNA therapeutics and small molecule drugs vie with each other. The effectiveness of each modality depends on the molecular mechanism of the disease and the “druggability” of the target. An example of a disease where small molecule drugs have been highly successful is cystic fibrosis (CF). CF is an inherited disorder caused by mutations in the gene encoding the cystic fibrosis transmembrane conductance regulator (CFTR; Kerem *et al*, 1989; Riordan *et al*, 1989; Rommens *et al*, 1989). CFTR is an anion channel that helps maintain fluid‐ and electrolyte‐homeostasis in multiple organs (Knowles *et al*, 1983; Quinton, 1983; Rich *et al*, 1990; Kartner *et al*, 1991). Loss‐of‐function leads to the accumulation of excessively viscous mucus that obstruct passageways. In the lung, this leads to airway blockage and causes repeated cycles of bacterial infections and chronic inflammation. Lung disease and respiratory failure are currently the main causes of morbidity and mortality in patients (Shteinberg *et al*, 2021; Fig 6C).

The *CFTR* gene displays mutational heterogeneity; to date, around 2,000 *CFTR* variants have been identified in patients with CF and CFTR‐related disorders. These mutations are classed into six groups based on how they affect the production, trafficking, function or stability of CFTR (Welsh & Smith, 1993). Class II mutations, which interfere with CFTR trafficking to the cell surface, are the most common, with an in‐frame deletion of phenylalanine 508 (F508del) affecting more than 70% of CF patients.

For decades, therapies were palliative and focused on medications that reduce symptoms, such as mucolytics, anti‐infectives, and pancreatic enzyme replacement. The development of small molecule modulators of CFTR that promote channel trafficking or function revolutionized CF treatment (Cutting, 2015). Nearly 90% of people with CF stand to benefit from these drugs, which are typically used in combination. There are, however, subsets of patients who carry *CFTR* mutations that lead to a complete loss of the protein, precluding the effective use of CFTR modulator therapy.

RNA‐based therapies might fill this gap. ASOs with different mechanisms of action such as inhibition of nonsense‐mediated mRNA decay or *CFTR* splice modulation are currently in pre‐clinical development (Kim & Krainer, 2023). These ASOs are often mutation‐specific and may therefore be suitable for only a small subset of CF patients. Earlier efforts to commercially develop ASOs for CF therapy have met with limited success. Development of eluforsen, an ASO that aims to insert the three missing bases in the F508del CFTR mRNA through an unknown mechanism, has recently been suspended, despite showing potential to improve lung function in homozygous F508del CF patients (Sermet‐Gaudelus *et al*, 2019; Drevinek *et al*, 2020). This ASO targets a similar patient group as the highly successful triple‐drug combination of the small molecule modulators ivacaftor, elexacaftor, and tezacaftor.

Gene replacement therapy allows treatment of all CF patients, regardless of the underlying genetic mutation. This approach has been explored since shortly after the identification of *CFTR* as the CF gene (Zabner *et al*, 1993). While initial clinical trials using AAV‐mediated gene delivery did not produce major improvements in symptoms (Wagner *et al*, 2002), a new therapy based on advanced vector engineering is currently in phase 1/2 clinical trials (NCT05248230). Delivery of CFTR mRNA as an alternative to DNA‐mediated gene replacement is actively pursued as well, with mixed results so far (Fig 6C). After encouraging initial reports, MRT5005, a drug that delivers CFTR mRNA packaged into lipid nanoparticles as an inhalable aerosol, failed to show improved lung function during the second interim analysis of the phase1/2 clinical trial data (NCT03375047), according to company statements. Nonetheless, MRT5005 was well‐tolerated, and the trial will continue.

CF is also amenable to CRISPR‐based genome editing approaches. Experiments in patient‐derived organoids have shown that allele‐specific corrections of aberrant *CFTR* splicing (Maule *et al*, 2019) and base editing to correct specific mutations within the *CFTR* gene (Geurts *et al*, 2020) are feasible. Still, to achieve durable therapeutic effects, the disease‐causing mutations must be corrected within lung stem cells, and targeted delivery of the therapeutic modalities to these cells remains a major challenge.

## Outlook

Advances in medicinal chemistry, a better understanding of the versatile cellular functions of RNA, and the experiences gained from decades of preclinical and clinical studies have established a strong foundation for the future of RNA therapeutics. Continuous optimization of RNA drug design, be it ASOs, siRNA drugs, mRNAs, and CRISPR‐Cas editing tools, will improve drug efficacies. An impressive illustration of this concept is the improvement in metabolic stability, durability, and potency of siRNA therapeutics achieved through advanced chemical modifications. For example, the prototype GalNAc–siRNA conjugate revusiran, which targeted transthyretin mRNA for the treatment of hATTR, was metabolically labile and therefore required high and frequent dosing. The drug was poorly tolerated in a phase 3 clinical trial and its development was discontinued. Optimized chemistry led to siRNA drugs with a satisfying safety profile and increased potency, facilitating dosing as infrequently as once every 6 months (Ray *et al*, 2023). Likewise, RNA drugs that exhibit limited efficacy in current clinical trials can serve as the developmental basis for next‐generation drug candidates.

Despite the spirit of optimism, RNA therapeutics face remaining challenges, including economic ones. High manufacturing and regulatory costs and the expense of clinical trials combined with the limited size of the potential market in cases of rare diseases might mean that the retail price charged for a treatment, if it is developed, may be unaffordable for most patients. Another major problem that currently stands in the way of achieving broad applications for RNA therapeutics is targeted *in vivo* delivery. For example, most ASO therapeutics to‐date use local delivery to specific sites, e.g. the eye or spinal cord, or delivery to the liver. It is expected that optimized combinations of RNA chemical modifications, conjugation with cell‐targeting ligands, and improved nanoparticle carrier systems will enhance the efficiency of RNA drug delivery and enable therapeutic molecules to reach previously inaccessible target tissues.

Apart from the RNA therapeutic modalities discussed in this review, there are others in preclinical and clinical development. These include ASOs or siRNA drugs that activate gene expression (Li *et al*, 2006; Janowski *et al*, 2007; Liang *et al*, 2016, 2017; Johnson & Corey, 2023), tRNAs that have been re‐coded to facilitate read‐through of nonsense mutations (Porter *et al*, 2021), and CRISPR interference and activation systems. The latter are comprised of a catalytically inactive Cas9 mutant fused to either a transcriptional repressor or a transcriptional activator, respectively (Gilbert *et al*, 2013, 2014; Maeder *et al*, 2013; Perez‐Pinera *et al*, 2013). Aptamers – short structured RNAs with high specificity and affinity for target molecules – can be used to modulate these targets or employed as carriers for delivering other therapeutic agents to specific cells or tissues (Zhou & Rossi, 2017).

While current clinical RNA therapeutics all target eukaryotic cells, RNA drugs can also be applied to prokaryotes. Proof of principle of the efficacy of ASOs in eliminating diverse bacterial species by targeting essential bacterial genes at the mRNA level has been established, including in animal models (Good *et al*, 2001; Daly *et al*, 2018). This highlights opportunities for the development of programmable RNA antibiotics to combat multidrug‐resistant pathogens (Sully & Geller, 2016; Vogel, 2020).

Looking ahead, we expect to see developments in all areas of RNA medicine in the near future – the possible benefits for patients are too great to ignore.

## Author contributions

**Jörg Vogel:** Conceptualization; writing – review and editing. **Anke Sparmann:** Writing – original draft; writing – review and editing.

## Disclosure and competing interests statement

JV is a member of the Advisory Editorial Board of *The EMBO Journal*. This has no bearing on the editorial consideration of this article for publication.
